# Adaptive capacity and flexibility of the Neanderthals at Heidenschmiede (Swabian Jura) with regard to core reduction strategies

**DOI:** 10.1371/journal.pone.0257041

**Published:** 2021-09-07

**Authors:** Berrin Çep, Benjamin Schürch, Susanne C. Münzel, Jens Axel Frick

**Affiliations:** 1 Department of Early Prehistory and Quaternary Ecology, Institute of Prehistory, Early History and Medieval Archeology, University of Tübingen, Schloss Hohentübingen, Tübingen, Germany; 2 Institute for Archaeological Science, Archaeozoology, University of Tübingen, Tübingen, Germany; Universita degli Studi di Ferrara, ITALY

## Abstract

The branched reduction system at the Heidenschmiede described here is hitherto exceptional for the Middle Paleolithic of the Swabian Jura. By means of refits and supporting objects, we are able to describe a superordinate reduction system that combines several individual reduction concepts, such as Levallois and blade production, within one volume. In the Middle Paleolithic of the Swabian Jura, blade technology has thus far played a rather minor role. On the one hand, it is possible to split a selected volume (nodule) into three parts, which are reduced separately according to individual concepts. On the other hand, it is also possible to reduce parts of a volume with one concept first and then with another. The hypothetical reduction system can be branched or linear, thus emphasizing the technological flexibility in core reduction, which requires a high degree of cognitive skills of three-dimensional imagination.

## Introduction

The stone technology of the Middle Paleolithic is characterized by a variety of concepts and methods of core reduction. Most of the concepts for flake and blade production follow the principle of predetermination of the blanks, which is achieved by the configuration and preparation of the reduction face and striking platform or by the choice of the direction of percussion. The most detailed concepts of blank production are the various Levallois, Discoidal and Quina concepts and methods [1–15]. For French sites, there is a correlation between core concepts, time, regional distribution and the raw material economy, although a concurrent occurrence of different concepts is not ruled out and chronological differences in the spatial distribution can only be identified as a tendency [16]. Also, there is evidence of different usage of various core concepts over time in southern Germany. In the G-layers of Sesselfelsgrotte in the Franconian Jura, there is a progression from Quina methods to the recurrent centripetal Levallois method and to the recurrent parallel Levallois method [17]. For the Swabian Jura, lower Middle Paleolithic layers are also thought to be associated with the Quina concept, while upper Middle Paleolithic layers yielded Levallois and Discoidal cores [18].

Sometimes it is difficult to detect these systems, as a “fragmented character” [19] created by deposition conditions and the import and export of objects [20,21]. For example, lithic cores are not always completely reduced by means of a single concept, but occasionally entire cores or parts of them were further reduced within another concept. Furthermore, blanks were also processed into cores. These highly dynamic processes on tools were first described by S. Krukowski as Pradnik cycle [22,23]. The reuse of lithic objects is considered as one of the characteristics of the Middle Paleolithic [24–29].

**Fig 1:**
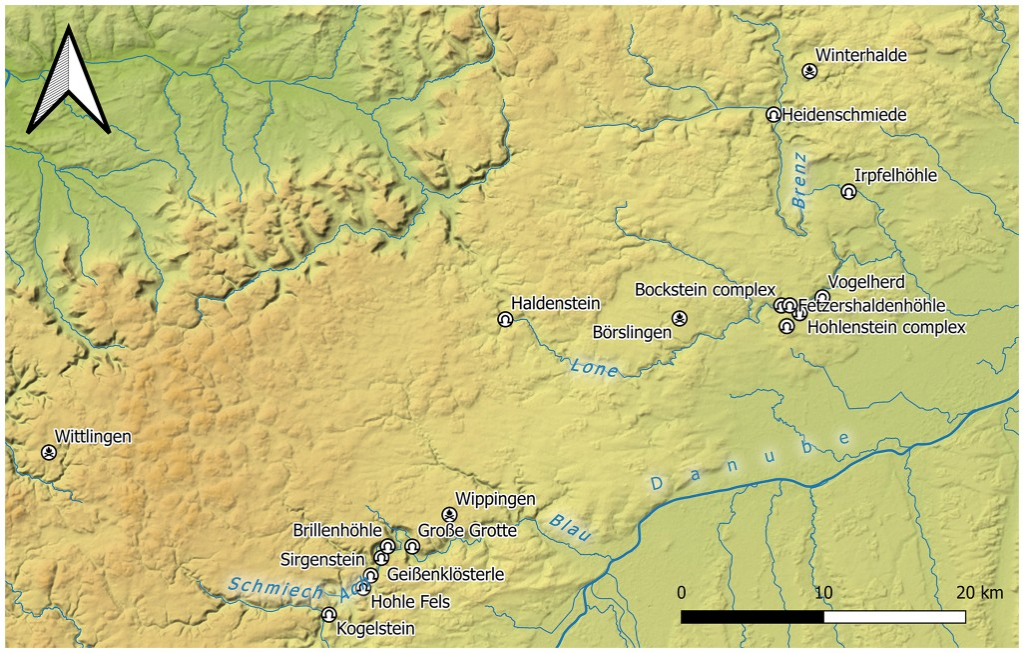
Map of Middle Paleolithic sites of the Swabian Jura discussed in the text. Legend: Caves/Abris and open-air sites are marked with a respective symbol. The site Heidenschmiede is in the upper right corner. Background map: C. Summer (ROCEEH), this work is distributed under CC-BY 4.0 license, see doi.org/10.5281/zenodo.3460300.

As early as the last century, it was noted that the Middle Paleolithic assemblages of southern Germany are not typologically homogenous and thus cannot be classified into established units, e.g., Mousterian or Micoquian, in their entirety [18,30–41]. The assemblages mainly originate from old excavations carried out at the beginning of the last century. As a result of the poor documentation of these excavations, the finds are often decontextualized due to the lack of stratigraphic, spatial and radiometric data. Furthermore, considering the long period of time the Middle Paleolithic of the Swabian Jura lasts—at least since the beginning of the last glacial period [39,40,42]–there are only few documented sites (see Fig 1). For these reasons, a complete chronological framework for the region remains wanted. In addition, the dominance of cave sites provides a limited picture of the composition of assemblages, which may be different for open-air sites with potentially different site functions. Hence, it is difficult to recognize a structure within and between the assemblages and the assemblages thus appear variable.

The low mountain range of the Swabian Jura in southwestern Germany ranges from the Hegau in the southwest to the Nördlinger Ries in the northeast. Its northern part extends to the *Albtrauf* (steep northern Swabian Jura) and in the south it is bordered by the Danube. It is particularly in the caves of this karst region that Paleolithic and Mesolithic sites have been preserved (see Fig 1). The Middle Paleolithic layers were also found mostly in the cave sites along the river valleys, while stratified open-air sites are rare or documented only by isolated surface finds [43–47]. The cave sites are concentrated in the following valleys: Lauchert valley (western part of the Jurassic Mountain range); Blau, Ach and Schmiech valley (central part) and Lone valley (eastern part). The number of known open-air sites is extremely small (list of sites in S1 Table).

**Fig 2:**
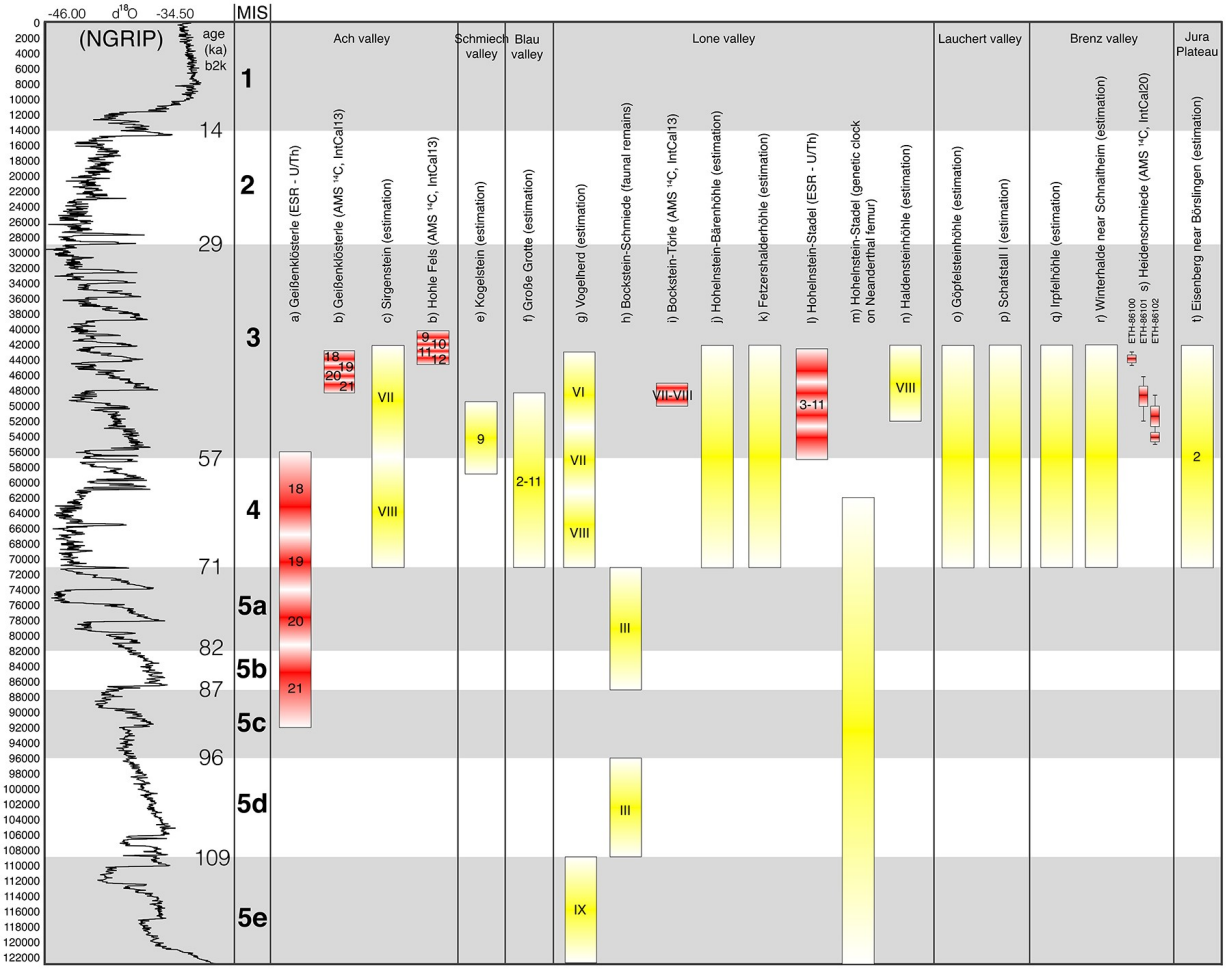
Radiometric dating and chronological classification of the Middle Paleolithic of the Swabian Jura. Legend: Oxygen isotope according to NGRIP [80], Greenland interstadials according to Rasmussen et al. [81]; a) Geißenklösterle (ESR—U/Th) [70], b) Geißenklösterle (AMS ^14^C) [75], c) Sirgenstein (estimates) [41], d) Hohle Fels (AMS ^14^C) [82,83], e) Kogelstein (estimates) [51], f) Große Grotte (estimates) [84], g) Vogelherd (estimates) [41,42], h) Bockstein-Schmiede (Fauna) [52], i) Bockstein-Törle (AMS ^14^C) [73,74], j) Hohlenstein-Bärenhöhle (estimates) [85], k) Fetzershalderhöhle (estimates) [86], l) Hohlenstein-Stadel (Fauna and Sediment) [76], m) Hohlenstein-Stadel (genetic clock from Neanderthal femur) [79], n) Haldensteinhöhle (estimates) [87], o) Göpfelsteinhöhle (estimates) [88,89], p) Schafstall I (estimates) [88,89], q) Irpfelhöhle (estimates) [90], r) Winderhalde bei Schnaitheim (estimates) [91], s) Heidenschmiede (AMS ^14^C, this study and Münzel & Çep in press) and t) Eisenberg near Börslingen (estimates) [43,46,92]. Red bars—radiometric dating (ESR—U/Th and AMS ^14^C) and yellow bars—estimates.

The chronology of the Middle Paleolithic of the Swabian Jura is mainly based on stratigraphic correlations and limited radiometric data (see Fig 2). The beginning is estimated within the Eemian and the following last interglacial [39,40,48], as evidenced by a single molar of a straight tusk elephant from the bottom layer AH IX at Vogelherd dated to the Eemian [42,49,50]. However, the sediments of most of the cave sites are younger than the last interglacial. This is also due to geological processes and studies have shown how these processes influenced the sediments in the valleys [39,49,51–69]. The latest Middle Paleolithic occupation took place in the Ach valley approximately between 45–42 ka cal. BP, according to the dates from Geißenklösterle and Hohle Fels [41,70–75]. The ^14^C-dates between 47 and 43 ka cal. BP from one of the Middle Paleolithic layers of the Hohlenstein-Stadel in the Lone valley also testify an occupation at the end of the Middle Paleolithic [76–78]. However, it must be kept in mind that the Neanderthal femur from this site was dated by the genetic clock (mutation rate) to about 124 ka BP and is to be placed in MIS 5 [79]. At Kogelstein the finds belong to an interstadial before the Hengelo but after the Odderade interstadial [51]. Fig 2 presents an overview of the chronological classification of the Middle Paleolithic of the Swabian Jura, based on radiometric data and estimates currently available.

The Middle Paleolithic assemblages of the Swabian Jura can be grouped on the basis of the existing reduction strategies [18] and the outlined chronological framework (see Fig 2) so as to enable the identification of chronological tendencies. On the one hand, there are assemblages with Levallois and Discoidal reduction strategies, used primarily for flake production, at the very end of the Middle Paleolithic (late MIS 4 to early MIS 3). These include the assemblages from Sirgenstein [93], Kogelstein [51], Hohlenstein-Stadel and Hohlenstein-Bärenhöhle [76,85, but see also 79], Geißenklösterle [94,95] as well as Große Grotte [84,96]. On the other hand, there are older assemblages, like Bocksteinschmiede [52,97–100] and, possibly, Heidenschmiede (this study and [101]), with cores that were reduced according to very specific reduction strategies combining features of Levallois, Quina or blade reduction. At Bocksteinschmiede, aspects of Levallois and Quina reduction are combined. This reduction provides blanks for backed and naturally backed blanks, as well as for several Keilmesser [98].

A different combination of reduction concepts is found at Heidenschmiede, combining aspects of Levallois and blade production on different reduction faces. So far, blade production is rare in the Swabian Jura—Heidenschmiede is an exception in this respect. Besides Levallois blade cores there are also non-Levallois blade cores in the assemblage. One of these cores very descriptively documents a particular process by reusing a broken core with a different reduction concept.

This process is illustrated by means of refits and is shown in this paper. The example from Heidenschmiede gives new insights to these highly dynamic processes of such “fragmented” assemblages and the reuse/recycling of objects. Thus, they help answer the question: Are there individual concepts that stand alone or is there a superordinate concept with multiple core concepts?

The fact, that the blade technology is known since the last interglacial in Central Europe raises the question of why it can be evidenced at the Heidenschmiede for the first time.

### Heidenschmiede

**Fig 3:**
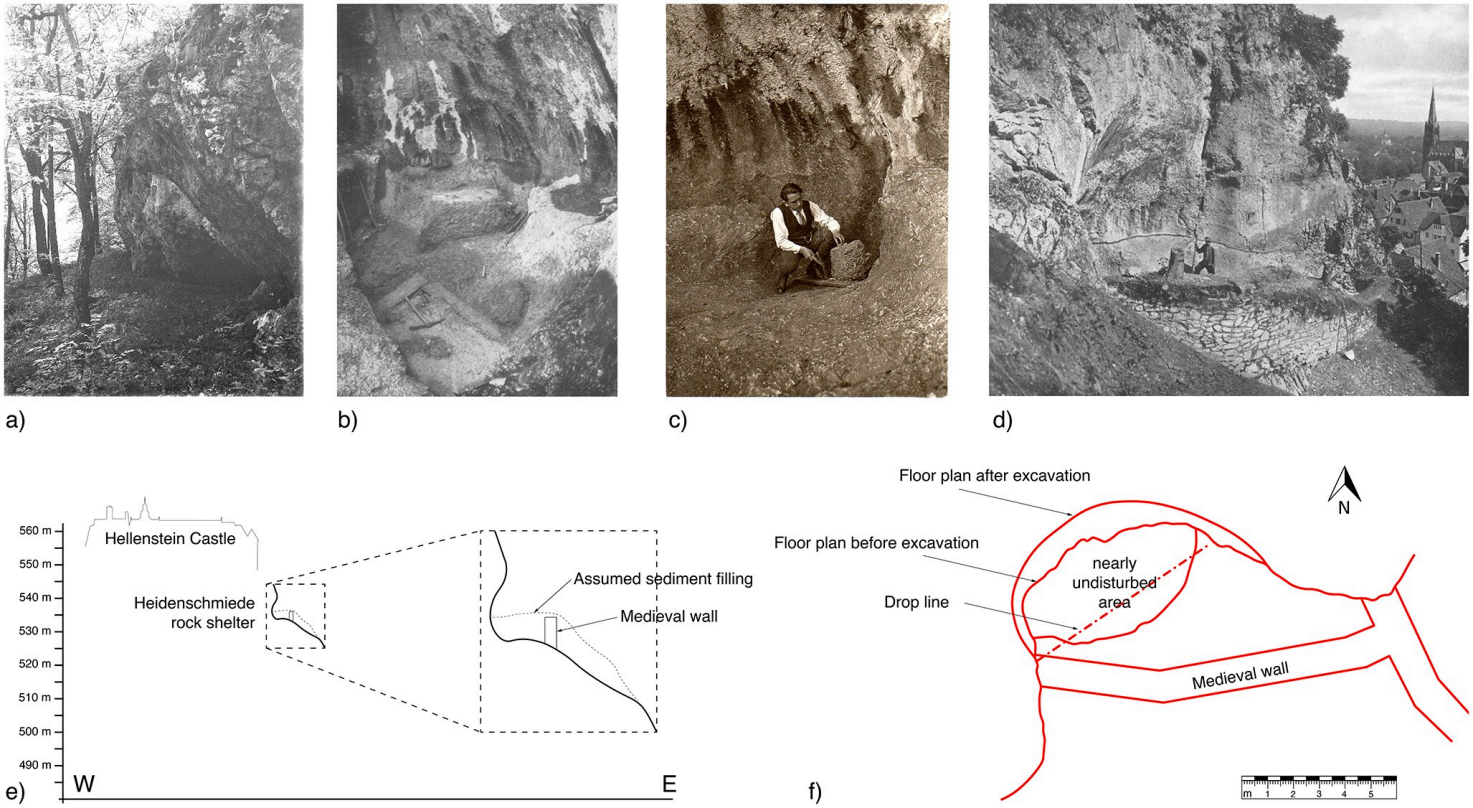
Images of Heidenschmiede. Legend: a) Rock shelter before excavation in 1930 (G. Odenwald); b) Rock shelter during excavation in 1930 [103]; c) Hermann Mohn during excavation in 1930; d) Rock shelter with medieval wall during excavation in 1930 [103: Taf. III1]; e) Altitude of the Hellenstein castle and the Heidenschmiede (section along the slope at Heidenschmiede redrawn from [103: Taf. II2]) and f) floor plan of Heidenschmiede, redrawn from [103: Taf. II1].

Heidenschmiede is a rockshelter, just below the castle of Hellenstein in the city of Heidenheim at the Brenz river in the eastern part of the Swabian Jura (see Figs 1 & 3). The site was discovered in 1928 [102] and excavated in 1930 [103,104] by the amateur research H. Mohn. The stratigraphy was strongly disturbed by a medieval stone wall. For this reason, Mohn did not pay attention to the stratigraphic position of the finds, although in small areas he was able to establish a stratigraphic order. In the same year, E. Peters was commissioned by the Württemberg State Office for Cultural Heritage (*Württembergisches Landesamt für Denkmalpflege*) in Stuttgart to finish the excavation. However, due to the thorough prior excavation, there was hardly any sediment left. Due to the lack of stratigraphic information, Peters reconstructed and published the chronological sequence using tool types [103].

He assigned the tool types to the Acheulian and the Moustérien, whereby he also recognized pieces of “microlithic character”, which were later considered by E. Wagner [105] as little blanks or chips. Because of the disturbance by the wall, the layer observation of Mohn was not considered useful [105]. Riek [102] suggested the presence of a handaxe culture or Upper Acheulian. Subsequent random reviews of the lithic artefacts by H. Müller-Beck [32] and G. Bosinski [106], who also noted, that only the Micoquian was clearly recognizable, attested the finds to be exclusively of Middle Paleolithic origin from several horizons.

Since the archaeological remains were never completely analyzed, the fauna and the lithic material of the Heidenschmiede were currently reevaluated by S. Münzel und B. Çep [101]. The following is a brief summary of their results: The stone artefacts are predominantly made of locally available raw materials. The assemblage shows a wide range of Middle Paleolithic tools. The most abundant tools, besides retouched flakes and blades are various types of scrapers. There are only a few bifacial and unifacial tools. They include bifaces, bifacial and unifacial Keilmesser, as well as unifacial and bifacial points. In addition, there are several “Groszaki” (n = 8), an artefact type that Bosinski [106] formerly described as “*umlaufend perlretuschierte Abschläge Typ Heidenschmiede*” (circumferential semi-abrupt retouched flakes).

Despite of the lack of stratigraphy, some technological and typological diagnostic features, in comparison to the Middle Paleolithic of the region, provide an idea of the time periods probably represented. These features are the Keilmesser and Groszaki, which are known, for example, from the late Middle Paleolithic of Central and Eastern Europe [106–115]. The nearest occurrence is known from Sesselfelsgrotte [17,116,117], Schulerloch [118,119], Schambach [120] or Große Grotte [84]. Other evidences of Groszaki were found in the remains of the Kleine Feldhofergrotte in the Neanderthal [121] or at Volkringshauser Höhle [122]. Unfortunately, the only radiometric data available from sites with such lithic elements are from the nearby Sesselfelsgrotte (G-layers), which clearly fall into the early MIS 3 [123,124].

The recent faunal analysis of Heidenschmiede is based on n = 3044 bone fragments with a total weight of almost 14 kg. 3.22 kg of which are charred [101]. The identifiable fauna revealed characteristic species for the Pleistocene Mammoth steppe environment [125], such as mammoth (*Mammuthus primigenius*), woolly rhino (*Coelodonta antiquitatis*), wild horse (*Equus ferus*) and reindeer (*Rangifer tarandus*). The faunal preservation of the majority of the bones are not very good, with the exception of the reindeer bones, which were generally better preserved and exhibited fine cut marks. This could have had chronological implications in that the better-preserved bones are younger. Consequently, Münzel and Çep decided to obtain radiocarbon dates and selected three bones with anthropogenic modifications, which were processed at the Laboratory of Ion Beam Physics, ETH in Zurich. The samples were a moderately preserved horse metatarsal with an impact mark (HDS-1), one well preserved but broken retoucher from a Bos/Bison metacarpal (HDS-2), and one well preserved reindeer femur shaft bearing scraping marks and an impact mark (HDS-4). The collagen preservation of all three samples was good for dating and the results place the Heidenschmiede assemblage into a Late Middle Paleolithic time range (see also Table 1 and s) in Fig 2), and is to be interpreted as an indication that an assignment of the fauna and the lithics to the Upper Paleolithic can rather be excluded.

**Table 1:** Radiocarbon AMS dates of three bones with anthropogenic modifications from Heidenschmiede.

Inventory Nr.	Sample Nr.	Sample Code	Description	Anthropogenic modification	^14^C age BP	±1σ	2 sigma range cal. BP	
							Lower	Upper
HDS 366	ETH-86100	HDS-1	Horse, metatarsal	Impact mark	40,882	612	42,937	44,684
HDS 438	ETH-86101	HDS-2	Bos/Bison, metacarpal	Retoucher	46,325	1,178	46,102	52,054
HDS 204	ETH-86102	HDS-4	Reindeer, femur	Impact & striations	48,732	1,583	48,577	54,980*

Legend: Calendar age 2σ range cal. BP-calibrated ranges. Calibrated with IntCal20 and Calib Rev 8.1.0 [126].

*Date most likely meets the end of the calibration data set.

## Material and methods

### Material

All find material of the archeological site Heidenschmiede is archived in three museums: Storage facility of the historic museums (*Historische Museen)* in Heidenheim, the Württemberg State Museum (*Landesmuseum Württemberg*) in Stuttgart and the Stuttgart State Museum of Natural History (*Staatliches Museum für Naturkunde Stuttgart*). These museums granted us access to the archaeological material of the site for analyses. The stone artifacts and faunal remains of the Heidenschmiede are currently being re-examined by B. Çep and S. Münzel. The preliminary results of this investigation were presented at the conference 15/16. 05.2017 Sélestat (France) “The Rhine During the Middle Paleolithic. Boundary or Corridor?” and a preliminary report was prepared [101]. A detailed investigation, especially of the stone artifacts is still in progress. The main refitting group presented in this paper comes from the storage facility of the Historische Museen in Heidenheim.

### Methods

All data used in this report are based on the ongoing work of Çep and Münzel. Here artifacts are examined according to the diacritical and metric methods generally used in Paleolithic Archeology to classify individual artifacts and artifact categories. These includes raw material determination, the observation of technological and technical features, the detection of external influences on the artefacts, typological determinations and metric analyses as well as refitting attempts to reconstruct production sequences. The core reduction processes shown by the case study discussed in this paper result from refitting attempts of the material.

In order to trace and represent the process of the technological concept available at Heidenschmiede, different methods of analysis were combined. The central object of this study was investigated by means of a working stage analysis in order to examine the successive stages and the branching—this was carried out using Bataille’s method [127,128], which is based on Pastoors [129–132]. Here, neighboring negatives are collected within on reduction step with a code. These steps are then interconnected to each other in order to be able to illustrate the working stages. Additionally, other raw material units were added where refits were possible (supporting objects). “*These refits also make it possible to determine the range of different techno-functional production strategies that are evident in different workpieces*” [128]. The combination of these two approaches allows the reconstruction of a cognitive process and a hypothetical reduction sequence, using only the few objects presented here.

**Fig 4:**
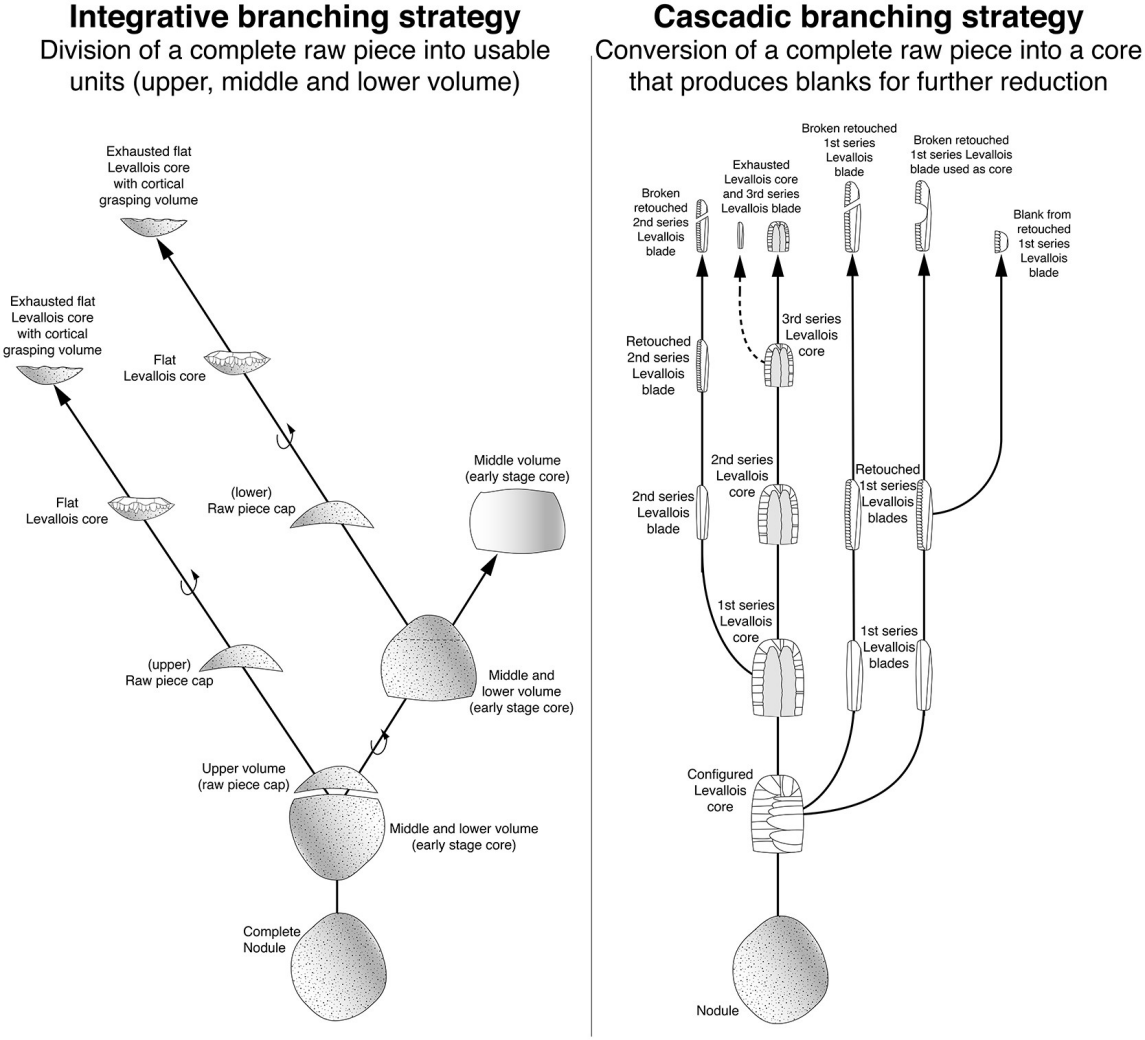
Diagram of integrative and cascadic branching strategies of lithic reduction. Legend: Two examples of branching strategies. Diagrams according to Geneste [135] and Bourguignon [29,134]. Left: Integrative branching strategy in which a complete volume (raw piece) is divided into usable units. In this case, an upper, middle and lower volume. Right: Cascade branching strategy in which a complete volume (raw piece) is converted to a core, which in turn can generate products for further use.

Based on the refits and the working stage analysis, which are the only feasible methods to detect these phenomena, the reuse and a branched process could be uncovered. For such branched lithic reduction processes, the term ramification is also used. Two strategies are described (Fig 4): 1. *type ramifié* [133] or integrative (branching) strategy [134] and 2. *type scalariforme* [135] or (cascadic) branching strategy [134]. These two strategies were first described by Geneste [133,135,136] for the Grotte Vaufrey and Les Tares sites. In the integrative strategy, an initial volume is split up into different sub-volumes, which can then be further processed. The cascadic strategy is structured in stages. In this strategy, detached products become cores, which in turn provide products for further use [29,134].

At Heidenschmiede, refits enable the discussion of a hypothetical and superordinate concept of a combined branched reduction.

Further processing of separate individual volumes is a maintenance process. This can take place immediately (reuse) or after an indefinite period of time (recycling). The difference between the two processes is based on the patination. If no differences in the patination of a piece are recognizable, then the assumption is that the manufacturing processes took place in a mutually temporally quite close framework. As it concerns a reuse and a further use of the piece. If differences are recognizable, then we assume a temporal gap, the piece is thus again integrated into the work process and recycled [see also the definition or reuse and recycling in 137]. In addition to the patina argument, the repeated use of very similar reduction sequences also speaks for an immediate reuse of the pieces.

## Results

Until now, it seemed that blank production in the Middle Paleolithic layers of the Swabian Jura was based on a number of lithic reduction concepts. In most layers, the reduction was performed using Levallois and/or Discoidal concepts, for example at Geißenklösterle [94,95], Große Grotte [84,96] or Sirgenstein [93]. This was regarded as one of the characteristics of the Middle Paleolithic of the Swabian Jura during MIS 4–3 [41,87]. Blanks were only occasionally produced using other concepts, like in Bocksteinschmiede [98], whereas blade production was quite rare.

In addition, not all cores at Heidenschmiede are clearly distinguishable as blade cores. This is primarily due to branched reduction sequences, which are characterized by the reuse of volume parts by changing from one reduction strategy to another. These branches and reuses are only discernible to a limited extent on individual pieces (cores and blanks). The only feasible method to detect these phenomena is refitting. At Heidenschmiede, refits enable the discussion of a hypothetical and superordinate concept of branched reduction.

### Core concepts at Heidenschmiede

Locally available raw material was used for all cores [101]. The core concepts can be divided into traditional categories, including Levallois flake and blade cores, unspecified flake and blade cores, polymorphic cores, one Discoidal core and individual cores of non-Middle Paleolithic character that may belong to the Neolithic period, considering the scarcely existing Neolithic pottery sherds. There are also some miscellaneous cores, including configured cores and fragmented cores (Table 2). In addition, there are tested cobbles.

**Table 2:** List of cores at Heidenschmiede.

Core		Number	Note
**Flake and blade core**	Residual recurrent Levallois core for flakes and blades	1	
	Flake and blade core	2	
	Flake and blade core, according to Boëda’s type D2	3	Example in Fig 10
	Configured flake and blade core, according to Boëda’s type D2	1	Example in Fig 10
**Discoidal core**		**1**	
**Core on flake**		**2**	
**Ventral core (Kombewa)**		**1**	
**Recurrent centripetal Levallois core**	Residual Levallois core	4	
	Flake and blade core of type D2 reused as Levallois core	1	Middle volume in Fig 5
**Configured Levallois core**	Recurrent centripetal Levallois core	2	
	Recurrent centripetal Levallois core on flake	1	
**Polymorphic core**		**2**	example in Fig 7.10
**Residual polymorphic core**		**4**	
**Blade core**		**1**	
**Bladelet and blade core**		**1**	
**Undetermined residual core**		**1**	
**Core debris**		**1**	
**Total**		**29**	

There are also cores that are difficult to classify into the common concepts, such as Levallois, Quina or blade cores. In most cases, these cores are a combination of all these core concepts. They all have two surfaces that are perpendicular to each other. The surfaces are alternately reduction and preparation faces. The upper faces have circulating blank scars, like recurrent centripetal Levallois cores; the lateral circumferential faces were often used for blade production. The blanks that are made from these cores are typologically Levallois flakes, *éclats débordants* (core-edge flakes), pseudo-Levallois flakes and non-Levallois blades. Some of the cores are severely exhausted, or sometimes the concept was changed during the reduction process, so that the remnant cores can no longer be assigned to a single concept, mainly due to the fact that at least two of the faces were reduced according to different specifications.

Similar to the Quina concept, the reduction was performed along two reference planes. However, no Quina target products were produced, but flakes with backs were mainly removed from the upper bulged face and blades were removed on the lateral face. According to Boëda’s core and reduction classification scheme [138–140] these are to be classified as belonging to type D.

### Branched production and reuse at Heidenschmiede: From non-Levallois blade core to Levallois flake core

**Fig 5:**
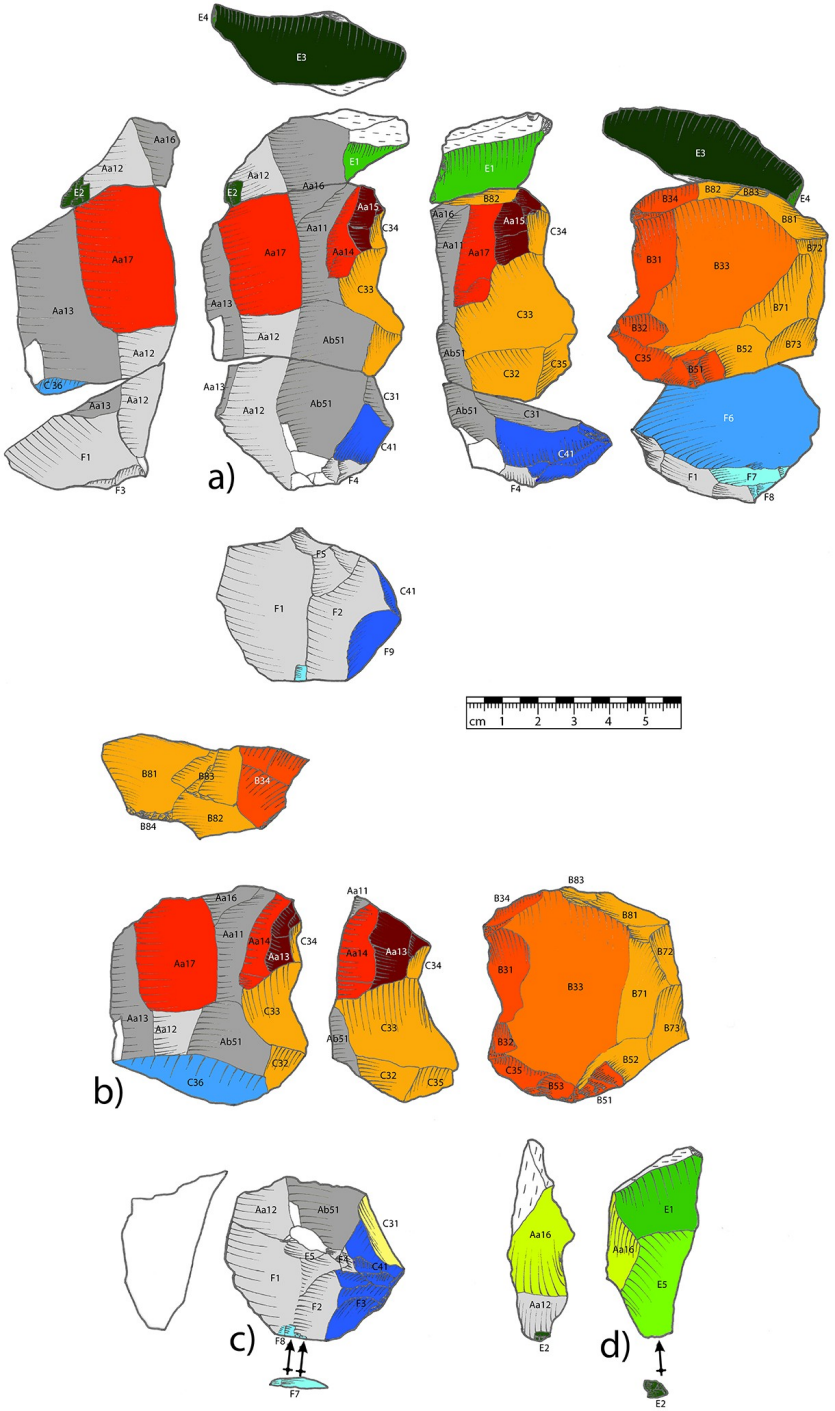
Drawing of the refitted reduction sequence. Legend: a) drawing of the three pieces, refitted; b) blade/Levallois core of the middle volume; c) core cap of the lower volume and d) core-edge blank of the upper volume. The bluish, reddish and greenish colored areas correspond to the working steps in Fig 6.

**Fig 6:**
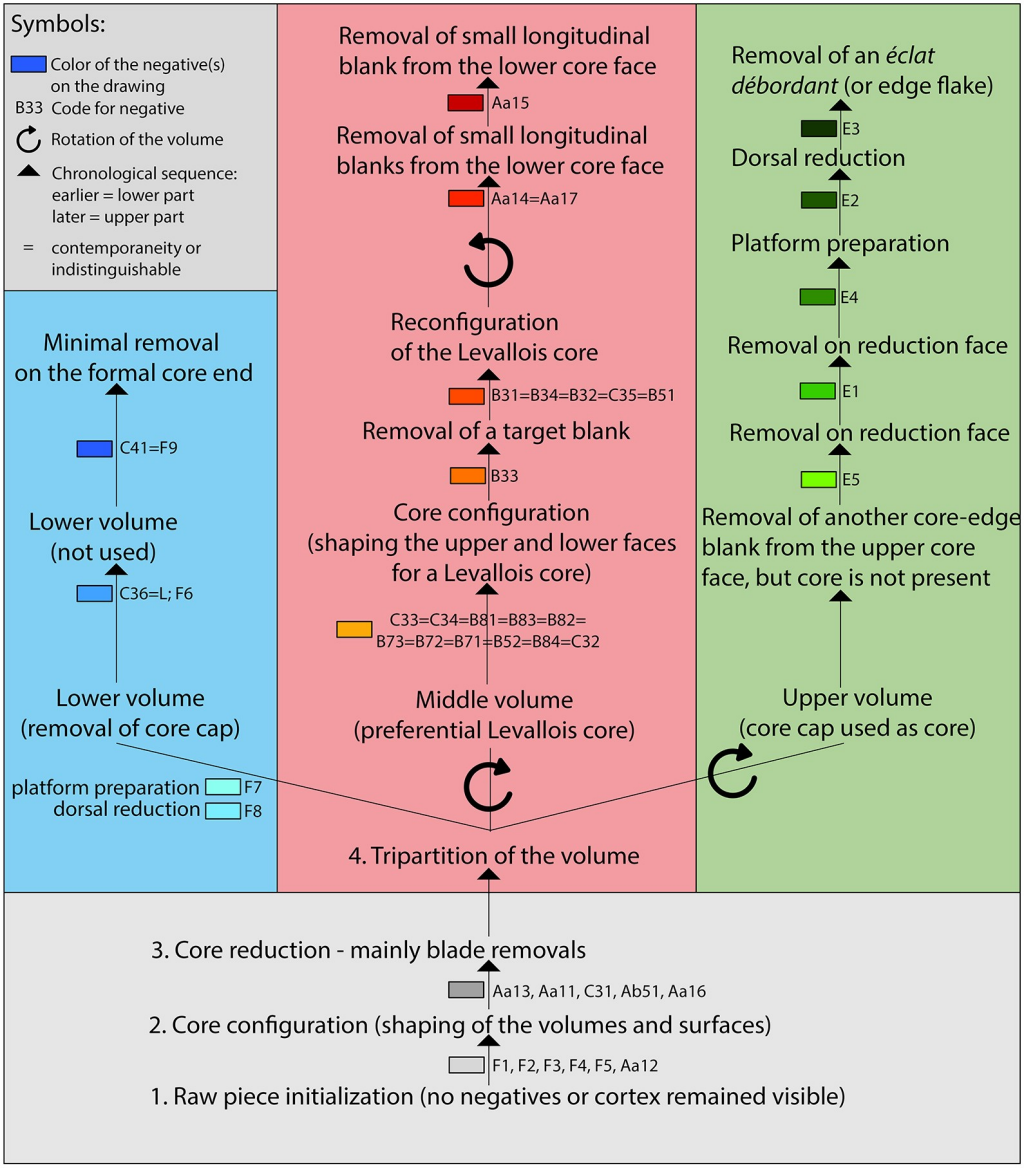
Working stage analysis of the three refitted pieces. Legend: Read from bottom to top, this working stage analysis represents the temporal succession of the reduction. After processing the entire volume (stages 1–3), the reduction splits up (stage 4). From this point on, the three parts (upper, middle and lower volume) go through different working stages. The symbols and colors used are specified in the figure.

The branched reduction sequence described here combines three core concepts that are traced by refits of three pieces (Fig 5). We thus conducted a working stage analysis (Fig 6) on these three refitted pieces (find numbers HS 23, HS 234 and HS 1587) according to Bataille’s method [127,128]. These three fragments reveal the unique adaptability and branching of blade and flake production in the Middle Paleolithic of the Swabian Jura. Due to the missing cortex, the initiation of the raw piece cannot be retraced. The first discernible step is the shaping of the volumes and the surfaces (F1, F2, F3, F4, F5, Aa12, see Fig 5). Subsequently, they were primarily blades (Aa13, Aa11, C31, Ab51, Aa16) that were removed bidirectionally from one reduction face. We identified a tripartition after the blade production. On the lower part of the core, a core-cap (bulged flake with centripetal negatives), which is a big flake removing the lower striking surface, was detached (C36, l, F6, F8, F7). On the lower part of the core, only minor modifications were conducted (C41, F9).

The upper part of the core was removed either intentionally (direct-hard-linear blow, or non-axial bipolar blow) or accidentally. There is only one *éclat débordant* (E1-E5), but the corresponding core is missing. This flake is the only proof of the existence of the missing upper part, which was used as a core-blank. This piece fits onto the upper part of the initial blade core and has two ventral surfaces. One of the ventral faces is the result of the separation of the former upper core part, the other ventral face is a result of the removal from the absent core. The core was reduced orthogonally to the former reduction surface of the blade core. After the removal of the upper and the lower part, the remaining central part was reused by reconfiguring it into a new core (C33, C34, B81, B83, B82, B73, B72, B71, B52, B84, C32).

Now, the reduction face of the former blade core becomes the bottom of the Levallois core. Vice versa, the bottom of the former blade core is configured into the reduction face of this Levallois core. For this purpose, a 180° rotation (along the longitudinal axis) and a 90° clockwise rotation (along the vertical axis) are performed. This process of rotation provides a suitable shape for Levallois reduction. The convexity of the piece was enhanced by the removal of several flakes (C32-35). After the configuration, a big target blank was removed (B33). The remnant core is a preferential Levallois core, which was subsequently reconfigured after (B31, B34, B32, C35, B51). The final step after the reduction of the Levallois core was the removal of small elongated blanks (Aa14, Aa17, Aa15) from the bottom of the core. This reduction may have been an attempt to once again remove elongated flakes from the former blade reduction surface. Based on the working stage analysis, we were now able to integrate the three refitted pieces into a hypothetical reduction sequence that illustrates the possibilities of reducing a volume as a single piece, dividing it, and further reducing the individual pieces.

### Supporting objects

This refitting sequence is backed up by objects that clarify the underlying concept. There are no cortical raw piece caps in the assemblage, so there are two possibilities for initiation: either the raw piece caps were removed off-site or these caps were used as core-blanks for further reduction. The detachment of the raw piece cap is illustrated by a refit that originates from non-axial bipolar reduction. It is reasonable to presume that when opening a nodule and removing a raw piece cap, the resulting area of the core was flattened by further bipolar reduction (see Fig 7.1 and 7.2). Another core can be interpreted as an attempt to form a Levallois core from a former blade core. Unfortunately, the reconfiguration of the core to a Levallois core was not successful and it was discarded prematurely (see Fig 7.3 and 7.4). The core cap, which can be removed at the lower end, is found both in the refitting sequence and as individual pieces (see Fig 7.5 to 7.7). If this core cap is not removed within the reduction, the middle and lower volumes are not separated from each other (Fig 7.10 to 7.12). Further reduction of this truncated cone on the upper bulged reduction face and lateral blade face results in a progressively decreasing core size (see Fig 7.8 and 7.9). There is a blade removal with a refit of a blade and a truncated cone-shaped core (Fig 7.10), and with another solitary truncated cone-shaped core (Fig 7.12). This concept is not an entirely unique case. All the pieces described above depict a concept frequently used at the site.

**Fig 7:**
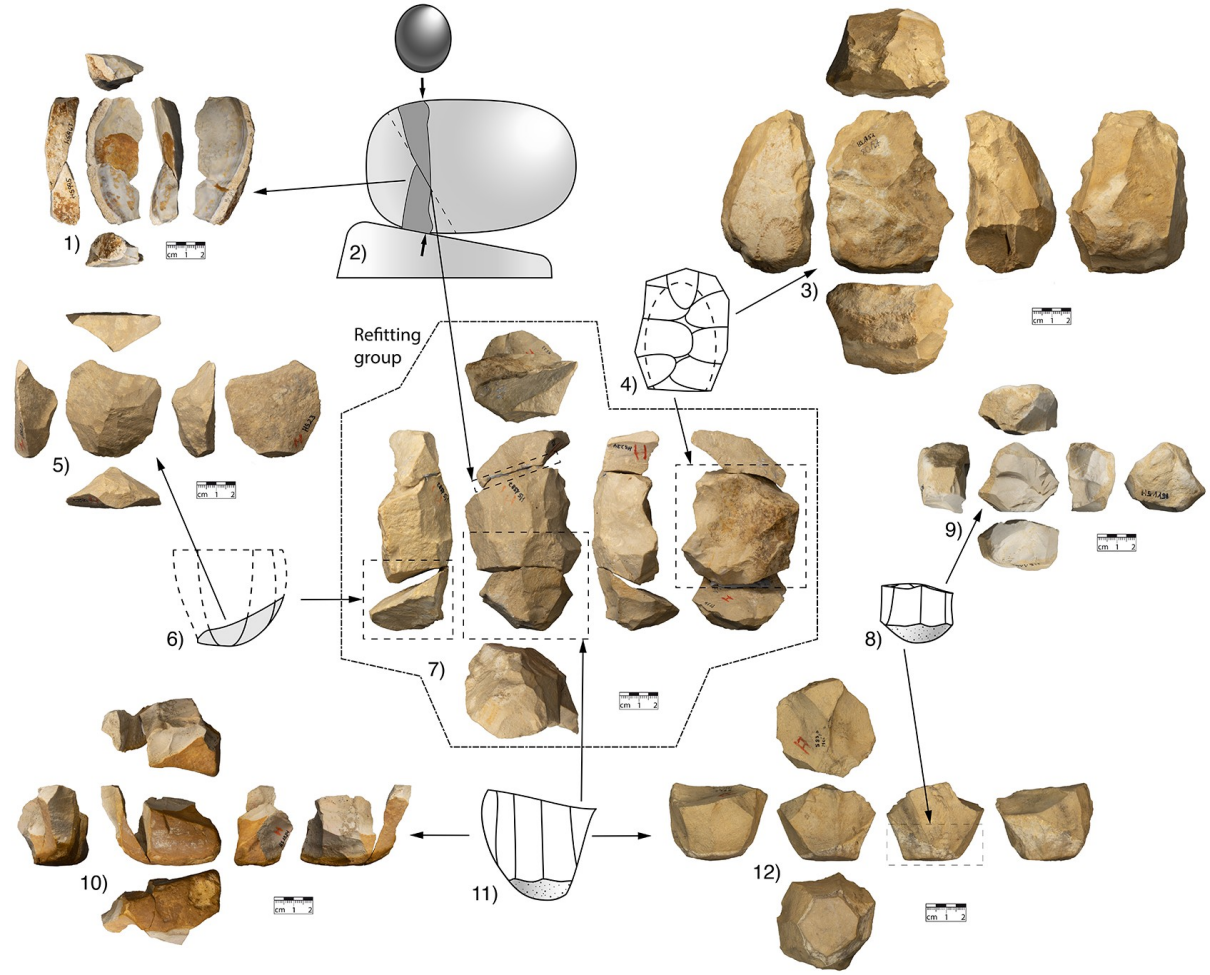
Supporting objects for the hypothetical reduction sequence. Legend: 1) refit of two flakes from non-axial bipolar splitting (inventory nos. HS 967 and HS 995); 2) diagram of bipolar splitting; 3) core with former blade negatives on one side and an unfinished Levallois reduction face on the other side (inventory no. HS 1851); 4) diagram of this blade/Levallois core, 5) lower core cap (bulged flake with centripetal negatives, inventory no. HS 23); 6) diagram of this core cap; 7) refitted reduction sequence of three pieces, as discussed (inventory no. HS 23, HS 234 and HS 1587); 8) diagram of a small depleted blade/flake core; 9) small depleted blade/flake core (inventory no. HS 1198); 10) refit of a small depleted blade/flake core and a lateral blade (inventory no. HS 31 and HS 1524; 11) diagram of a blade/flake core that is almost depleted and 12) blade/flake core that is almost depleted (inventory no. S 83,6 Hei). The pieces with inventory no. *HS* are all from the Storage facility of the historic museums (*Historische Museen)* in Heidenheim, the core with inventory no. *S83*,*6 Hei* is in the Württemberg State Museum (*Landesmuseum Württemberg*) in Stuttgart.

**Fig 8:**
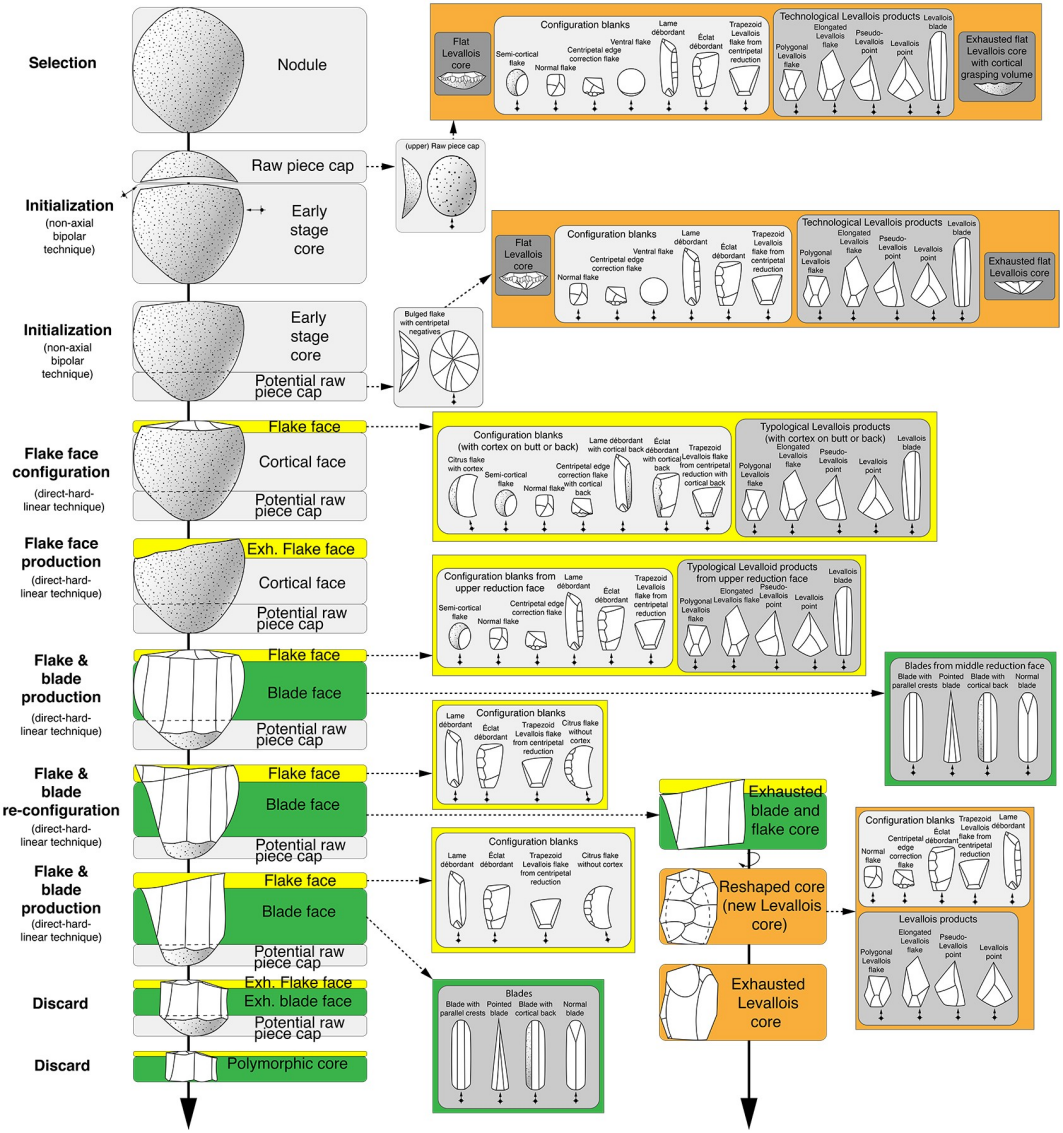
Reconstructed linear reduction sequence with added blanks. Legend: Hypothetical reduction sequence based on the mean volume and the products hypothetically generated in the individual reduction steps. The blade face is shown in green, the Levallois face in orange and the bulged flake face in yellow.

The reduction sequence documented here produced a variety of different blanks (see Fig 8). In addition to the raw piece caps and core caps of the upper and lower volume of the nodules already described, the flake, Levallois and blade reduction faces produced roundish-oval as well as polygonal and elongated blanks. The convexities on the flake and/or Levallois reduction face, in particular, resulted in different core-edge blanks and typological and technological Levallois target blanks, such as polygonal Levallois blanks or Levallois and pseudo-Levallois points. The blanks that modify the edge between upper bulged flake and lateral blade reduction face always show a distinct back, which can be found on the lateral edge (*éclats débordants*) as well as on the platform (core-edge correction blanks). The lateral blade reduction face produced blades of varying morphology.

## Discussion

### Towards a reconstructed reduction sequence

**Fig 9:**
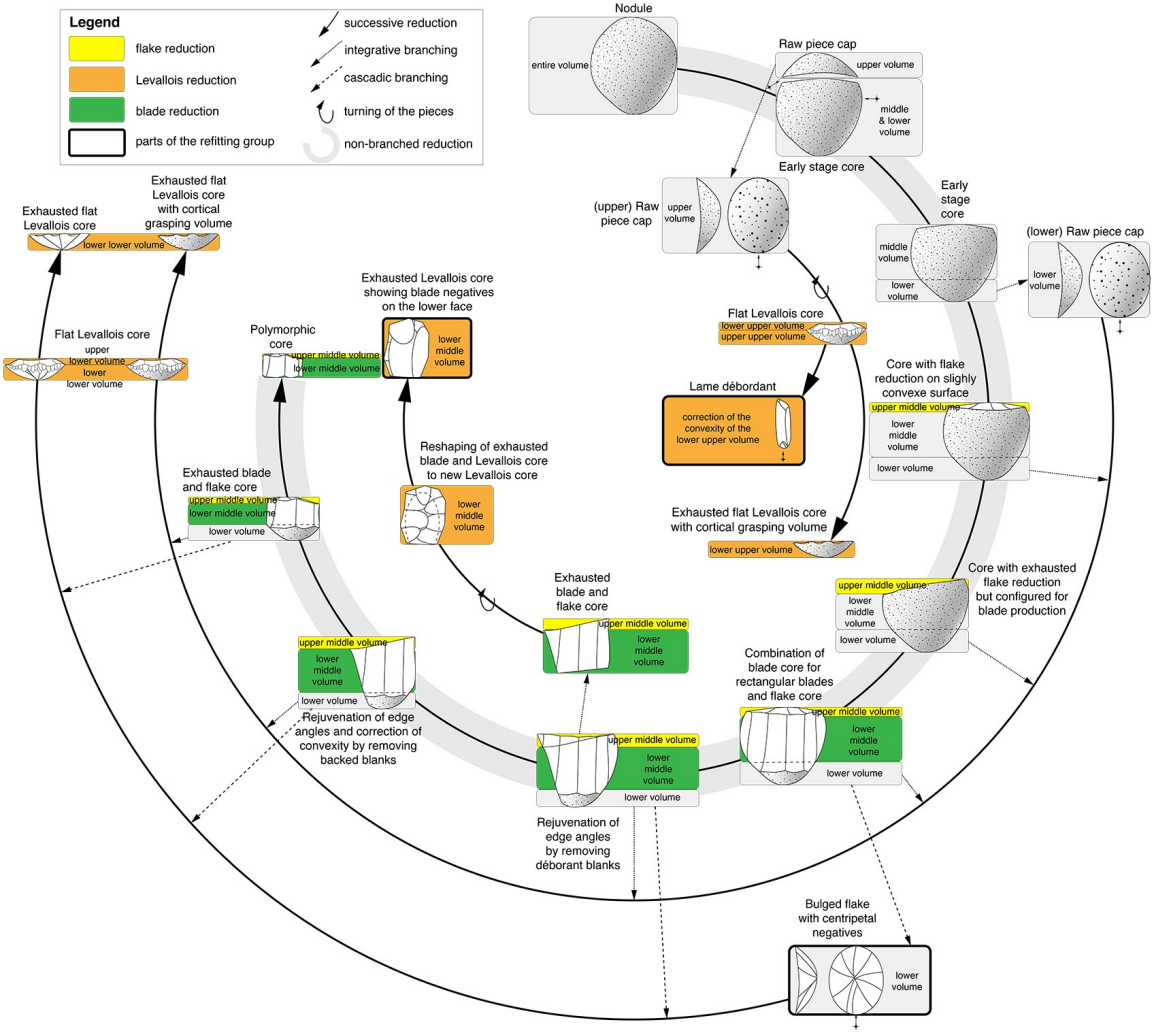
Schematic diagram of the reconstructed reduction sequence. Legend: The diagram contains all possible paths that can be followed with the reconstructed superordinate system. In addition to the non-branched reduction possibility, it contains the paths that can be taken through integrative and cascadic branching. The parts of the main refitting group are highlighted.

Combining the results of the working stage analysis of the refitted sequence (Figs 5 and 6) with the supporting objects (Fig 7), a diagram can depict branched reduction possibilities (Fig 9). After opening the nodule, the branching of the reduction already begins. A cortical raw piece cap (upper volume) and a (still) conjoined middle and lower volume are formed. The upper raw piece cap can now be transformed into a flat Levallois core. With the connected middle and lower volume, it is now possible to either make the upper surface convex or to remove a lower raw piece cap. The removed lower raw piece cap can in turn be shaped to a flat Levallois core. If this cap is not removed, it is possible to obtain both central blanks and core-edge blanks from the convex upper face successively, very similar as from a Levallois reduction face. The resulting core corresponds to the Type D2 scheme according to Boëda [140] for the production of blades.

After this face has been exploited, reduction can begin on the lateral flank. On the basis of one refitting sequence (Fig 7.7), the detaching of overlapping blades is likely to have started immediately. To maintain the edges between the reduction face and the striking platform, core-edge blanks are removed repeatedly. At this point, there are once again two possibilities for further reduction. Either the core is successively reduced on the upper face and the lateral flank so that a polymorphic residual core remains, or the core is reshaped extensively. In the case of the refitted reduction sequence (Figs 5 and 6), the lower volume (core cap) has been removed and the middle volume has been turned and rotated so that a Levallois core can be formed from the remaining volume. Based on the refitted reduction sequences (Figs 5 and 6) and the supporting objects (Fig 7), the underlying reduction concept becomes clear. The knapper must have been aware of this concept in order to be able to consider the possibilities of either further reducing the core or following one of the branching reduction paths in the respective stages of reduction.

The underlying concept not only implies that different reduction paths can be taken in certain stages. It also implies that the knapper is aware that she/he does not always have to execute each working stage in order to produce the desired products. It was thus possible to produce a very wide range of blanks. In addition to various typological and technological Levallois products (oval, polygonal or pointed blanks and blades) and bulged flakes (cortical and non-cortical), various blades could also be produced (see Fig 8). The scheme presented here implies that not every working stage and branch has to be executed in the reduction. They are merely possibilities. This can also be seen from the fact that some of the resulting core caps were not converted to Levallois cores (see Fig 7.5 and 7.7).

If the reconstructed reduction scheme is consistent with reality, this also implies that the reduction sequence may be terminated at a very early stage, when the desired blanks have been produced. Individual detached objects (e.g., the raw piece caps or the core caps) can be used as a basis for independent sequences (flat Levallois cores). The (cortical) lower raw piece cap, as well as the (non-cortical) core cap, can be removed at different times within the sequence. The medium volume, which was used for blade detachment, could similarly be rededicated. By rotating, it was possible to further reduce the volume by means of the Levallois concept.

### Branching

The conversion of volumes has been observed at many other sites [5,10,29,133–135,141–145], but most of them are based on converting blanks into cores, as defined by Bourguignon et al. [29], so we will only exemplify a few cases that are most similar to the branching strategy at Heidenschmiede. This phenomenon (converting blanks into cores), which mostly produces small products, has been described, e.g., for the southwest of France [134,144] and the Iberian Peninsula [141]. In contrast to this cascadic reduction, integrative reduction has been documented quite rarely (see also Fig 4). Apart from Heidenschmiede, the only examples we know are from Cantalouette IV [146], Vaufrey VIII [133], Orgnac 3 [134] and Barbas 1 [134]. However, the explicit linkage of both branching strategies is very difficult to identify in the literature. Such a linkage is most true for Cantalouette IV [146] and Combe Grenal niv. 29–30 [144]. In the first case, it was possible to refit a Levallois core with a conical blade core. The initial volume was thus divided into two parts, which were reduced separately with different concepts. In the second case, selected frost shards were configured into Levallois cores or blade cores. Blanks separated from these were in turn used as a matrix for further cores, which also provided usable blanks.

### Superordinate reduction system

**Fig 10:**
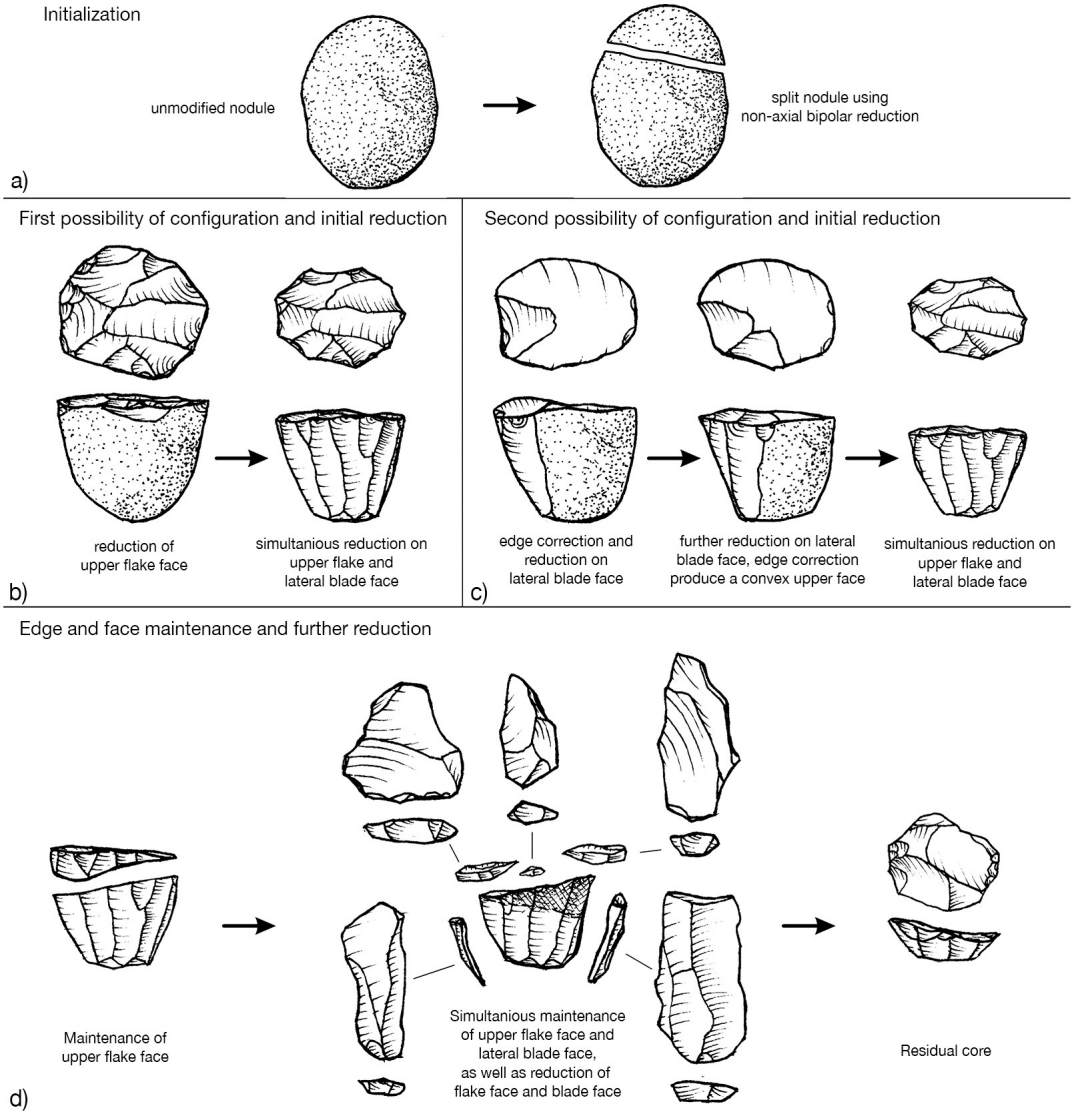
Reconstructed (almost) non-branched reduction sequence. Legend: Reconstructed nearly homothetic reduction on truncated cone-shaped core. Drawings by H. Würschem.

To return to the question formulated at the beginning: Are there individual concepts that stand alone or is there a superordinate concept with multiple core concepts? The reduction sequence of both integrative and cascadic branching presented for the Heidenschmiede is described according to Geneste [135] and Bourguignon [29,134]. In this case, both kinds of reduction branching are combined. The reduction shows—to some extent—products of cores which were reused as cores. The hypothetical scheme shows how different reduction concepts, previously considered individually, are interconnected in a highly dynamic system. The branched system at Heidenschmiede (Fig 9) includes cores, which are reduced using more than one individual concept. The minimally branched reduction system is gleaned from examples of refits and various cores (Figs 5, 6 and 7.9, 7.10 and 7.12). These examples clearly show how blades are removed from a truncated cone-shaped core and they also combine an upper convex Levallois-like flake face with a lateral blade face (Fig 10). The minimally branched and nearly homothetic reduction sequence proceeds according to the following hypothetical scheme: After opening an elongated, potato-shaped nodule with non-axial bipolar splitting (Fig 10a), there are two possibilities for configuration and initial reduction. In the first case, the upper face is bulged and used as a flake reduction face; in this case, the upper face and the lateral blade face are reduced simultaneously (Fig 10b). In the second case, an edge correction is performed first and subsequently the lateral blade face is reduced; after further reduction and successive bulging of the upper face, both faces are also reduced simultaneously (Fig 10c). Thicker edge-correction blanks are removed from the upper face to correct the angles and to maintain the faces. Once the core has been set up, it is possible to remove both blades and backed blanks simultaneously. This reduction sequence, branched within the early reduction phase, hypothetically produces quite flat, levalloid-appearing cores at the end of the sequence (Fig 10d).

The individual parts of the volume (upper, middle and lower volume) can be reduced according to independent concepts, when previously separated from the total volume. The upper and lower volume can be converted into Levallois cores. The middle volume can be reduced in different ways, depending on whether the lower volume is retained. If the lower volume is separated, the middle volume can also be converted into a Levallois core. If the middle and lower volume were not separated, the combination of flake and blade production is possible (see Figs 9 and 10).

According to Boëda’s scheme [138–140], there are three different reduction concepts combined in this superordinate reduction system proposed here: F3, F1 and D2, regardless of how much the reduction process is branched. In the case of the unbranched strategy, the reduction takes place according to the type D2. In the integrative strategy, the raw piece is divided into several usable volumes: 1^st^ Splitting of complete raw pieces (nodules) into a upper and contiguous middle and lower volume (**type F3**); 2^nd^ Utilization of the generated partial volumes by configuration into specific cores (upper volume into a Levallois core, **type F1**; middle volume into a Levallois core, **type F1** and lower volume into a Levallois core, **type F1**), if the middle and lower volumes are not separated from each other, the reduction can be carried out along two reference planes according to **type D2**. According to the cascadic strategy, it is possible to separate the lower volume later, after this part has also been freed from cortex. This may also apply to the upper volume. Subsequently, this flake can be configured to a Levallois core (**type F1**).

In addition to how the volume can be used, it is important to note the wide range of generated target blanks (Fig 8). In addition to the long narrow flakes and blades that can be detached from the lateral conical face, it is possible to detach both elongated blanks as well as shorter roundish or polygonal flakes from the upper flake face. The large number of backed blanks is particularly noteworthy (see Table 3).

**Table 3:** Lithic assemblage of Heidenschmiede.

Supporting objects	Number	Percentage
**Normal flake**	1005	45,64
**Cortical flake**	127	5,77
**Ventral flake (Kombewa)**	43	1,95
**Éclat débordant without cortical back**	263	11,94
**Éclat débordant with cortical back**	13	0,59
**Flake with cortical back**	82	3,72
**Citrus flake with cortex**	10	0,45
**Citrus flake without cortex**	1	0,05
**Primary crested flake**	1	0,05
**Secondary crested flake**	2	0,09
**Crested flake**	11	0,50
**Flake from surface working**	7	0,32
**Overshot flake**	5	0,23
**Rectangular flake (wider than long)**	2	0,09
**Rectangular flake**	1	0,05
**Core tablet**	2	0,09
**Core cap with negatives**	2	0,09
**Levallois flake**	122	5,54
**Levallois point**	14	0,64
**Pseudo-Levallois-Point**	73	3,32
**Flake detached from the terminal end of another flake**	1	0,05
**Flake from retouch**	1	0,05
**Normal blade**	151	6,86
**Blade with parallel negatives**	32	1,45
**Blade with cortical back**	6	0,27
**Primary crested blade**	3	0,14
**Primary crested blade with cortex**	3	0,14
**Primary crested blade with back from fissure**	2	0,09
**Secondary crested blade**	17	0,77
**Pointed blade**	14	0,64
**Overshot blade**	4	0,18
**Levallois blade**	14	0,64
**Burin bladelet**	2	0,09
**Core**	29	1,32
**Core debris**	4	0,18
**core tool**	3	0,14
**Tested pebble**	3	0,14
**Raw piece**	2	0,09
**Debris**	73	3,32
**Frost sherd**	17	0,77
**Heat debris**	7	0,32
**Undetermined object**	28	1,27
**Total**	**2202**	100,00

Legend: Half of the assemblage of the Heidenschmiede consists of normal flakes. Noteworthy are the high numbers of *Éclat débordants*, normal blades and Levallois flakes.

The back can be positioned on a lateral edge (core-edge blanks, *éclats* & *lames débordants*) or formed by a large butt, as is the case with pseudo-Levallois points (asymmetrical triangular or deltoid points) or blades that are not typologically considered Levallois blades. All these blanks are considered target blanks, as well as configuration and rejuvenation blanks, although it should be noted that the material has not yet been examined for traces of use-wear. Since these triangular and deltoid points are unretouched, we presume that these are informal tools that were used in exactly the same shape as they were detached. Peters named them hand points (*Handspitzen*) [103].

The question remains whether the further processing of the partial volumes of the reduction sequence discussed (Figs 5 and 6) is a reuse process taking place immediately or a recycling process taking place after some time [see also the definition or reuse and recycling in 137]. We presume that this is a process of reuse of the pieces rather than recycling, since the reduction sequence is not unique and numerous other pieces also indicate immediate further processing (see Fig 9). In addition, the concepts used at the site also exist by themselves independently of a ramified reduction system.

While it was still exceptional in the 1990s to find blade industries in a Middle Paleolithic context [147], well over a hundred sites are known today [148]. Révillion [149] was already able to show that there were other concepts for producing blades besides the Levallois blade technology. In Europe, blade technologies (both Levallois and Laminar) have been used since MIS 8 [147]. As with other technologies, the clear evidence for their presence increases strongly after the Eemian interglacial.

The blade cores and the aspect ratios of blanks, which indicate a high proportion of elongated flakes, illustrate that blades are an important component of the assemblage (see Table 3). This is unique for the Middle Paleolithic of the Swabian Jura [101]. There are blades in other assemblages, such as those from Große Grotte, Bocksteinschmiede or Kogelstein [31,51,96,98], but always in exceedingly small proportions.

## Conclusion

Based on physical and mental refits, this case study was able to demonstrate that the branched production and flexible application of the core reduction options were available in the cognitive “reservoir” [116] of lithic reduction concepts. Here concepts for the production of blades and other blanks played an important role. While the flow of the individual production sequences within a concept must be part of the individual concept reservoir, the interrelationship of the concepts and the possible branchings of production can also be part of the collective concept reservoir [see also 116,150,151].

The “concept reservoir” not only includes the individual lithic concepts, like Levallois and blade production, but also comprises the know-how of the application of these concepts to different surfaces within a volume. In addition, the knapper had to understand that parts of volumes can also be further processed using a different concept. This also includes the selection of surfaces for removing blanks with various morphologies. These skills require a keen three-dimensional imagination of the reducible volume and the knowledge of rotation processes to be applied during reduction. Similarly, the superordinate reduction system demonstrates the mental flexibility to recognize detached parts of the volume as additional usable volumes, visible, for example, in the use of raw piece caps and core caps.

The combination of individual concepts, as in Heidenschmiede, testifies to the “pronounced working memory” of the producers [see 152,153]. This includes the perception of the problems in knapping, different operational steps, as well as the understanding of a branched but nevertheless goal-oriented production. All in all, we can conclude that the branching of lithic production, combined with a sequence of distinct individual concepts, requires cognitive skills such as good spatial ability, three-dimensional imagination, creative thinking, manual dexterity and task-specific learning. This indicates both, mental flexibility and adaptive capacity.

Beyond the individual examination of cores, classified in categories and reduction concepts (cores and corresponding blanks), branched systems can only be recognized if their interplay is reflected in the assemblages. It is thus essential to look at assemblages with a holistic view [see 154, p. 165–166]. After all, individual reduction concepts can, but do not have to, be linked in a superordinate system.

The superordinate strategy, which could be reconstructed by the analysis of the refit-group, as well as the supporting objects, contains numerous individual lithic reduction concepts, which could be highlighted in the Swabian Jura [18,41,95,155] and thus shows the indirect simultaneity of these concepts. Whether this is an exceptional case or occurs more often cannot be clarified at the moment. This can, due to the strong branching of the reduction, only be clarified via refits. Due to the subsequent utilization of volumes, it is probably not possible in the majority of cases to carry out appropriate refits, as the pieces were subsequently heavily modified for use. Other potential reasons are: export of pieces or excavation circumstances.

So far, we cannot name any other sites in the Swabian Jura with such a combined integrative and cascadic branching strategy and this is surely due to the fact that refits were carried out only to a small extent. In earlier Middle Paleolithic assemblages of the Middle Paleolithic of the Swabian Jura like the Bocksteinschmiede, there is barely any Levallois technology [98]. At the end of the Middle Paleolithic in the Swabian Jura, there are sites where blanks were produced by means of Levallois and/or Discoidal technology, e.g., at Geißenklösterle [95] or the Hohlenstein caves [85]. These technologies are occasionally combined with a modest number of bifacial tools, e.g., Kogelstein or Große Grotte [18]. The role of bifacial technological strategies and especially the handaxes from Heidenschmiede has been overestimated by earlier researchers. However, non-bifacial technological strategies used at the site are far more common, as evidenced by several Levallois- and non-Levallois flake and blade cores and blanks. The assemblage of Heidenschmiede (see map in Fig 1) contradicts the temporal affiliation of the individual core concepts, because here, concepts are interwoven with each other. Research in recent decades has clearly shown that the reduction concepts identified in the Middle Paleolithic rarely stand alone, but are integrated into dynamic reduction systems [156].

Each site contains a snapshot of the entire reservoir of concepts [20,21,96,116,137]. This makes a chronological classification of the individual core concepts difficult. It is clear to us that the stratigraphic context and the ascription of the finds from Heidenschmiede to a temporal unit is not-conclusive. However, based on the refits, we were able to show that different concepts are simultaneously present within a nodule (Figs 5 to 10). This means that there must have been a broad reservoir of reduction concepts from which to draw flexibly. This could explain why a well-known concept such as blade production could only be found to such a high degree at Heidenschmiede. The concurrent existence of various reduction strategies, as well as their diversity and flexibility in use, are characteristic for the late Middle Paleolithic of the Swabian Jura. The mental capacity of Neanderthals is manifested not only in the use of various reduction concepts, but also in the individual and/or branched use of these concepts as appropriate to the situation.

## Supporting information

S1 Table List of sites on the Swabian Jura with evidence of Middle Paleolithic occupation. Legend: Sites and evidence, according to many authors: [31,32,43,44,46,48,51,52,70,76,84–86,88,89,91–95,97–99,106,157–176].
(ZIP)
