# Supplementary material for: Adaptive capacity and flexibility of the Neanderthals at Heidenschmiede (Swabian Jura) with regard to core reduction strategies
Source: PLoS One. 2021 Sep 7;16(9):e0257041. doi: 10.1371/journal.pone.0257041 (PMC8423277; doi:10.1371/journal.pone.0257041)
Supplement: S1 Table — Legend: Sites and evidence, according to many authors: [31,32,43,44,46,48,51,52,70,76,84–86,88,89,91–95,97–99,106,157–176]. (ZIP) [file pone.0257041.s001.zip › S1 Table.pdf]

Geographic area of the sites	Sites
**Western part of the Jurassic mountain range, in the Lauchert valley near Veringenstadt**	Göpfelsteinhöhle
	Schafstall I & II
**Central part of the Jurassic mountain range, in the Blau, Ach and Schmiech valley**	Kogelstein
	Sirgenstein
	Hohle Fels
	Geißenklösterle
	Große Grotte
**Central part of the Jurassic mountain range, near Bad Urach**	Wittlingen
**Eastern part of the Jurassivmountain range, in the Lone valley**	Hohlenstein with Stadel and Bärenhöhle
	Site of the Bockstein complex
	Fetzersladenhöhle
	Vogelherd
	Haldensteinhöhle
**Eastern part of the Jurassivmountain range, near the Lone valley**	Börslingen
**Eastern part of the Jurassivmountain range, in the Brenz valley**	Heidenschmiede
	Irpfelhöhle
	Schnaitheim
